# Heart rate detection properties of dry-electrode ECG compared to conventional 3-lead gel-electrode ECG in newborns

**DOI:** 10.1186/s13104-021-05576-x

**Published:** 2021-05-01

**Authors:** Hanne Pike, Joar Eilevstjønn, Peder Bjorland, Jørgen Linde, Hege Ersdal, Siren Rettedal

**Affiliations:** 1 Department of Paediatrics, Stavanger University Hospital, Post Box 8100, 4068 Stavanger, Norway; 2 Department of Research, Laerdal Medical, Stavanger, Norway; 3 Faculty of Health Sciences, University of Stavanger, Stavanger, Norway; 4 Critical Care and Anaesthesiology Research Group, Stavanger University Hospital, Stavanger, Norway

**Keywords:** NeoBeat, Electrocardiogram, Dry-electrode ECG, Heart rate, Resuscitation, Validation, Newborn, Neonate

## Abstract

**Objective:**

To compare the accuracy of heart rate detection properties of a novel, wireless, dry-electrode electrocardiogram (ECG) device, NeoBeat®, to that of a conventional 3-lead gel-electrode ECG monitor (PropaqM®) in newborns.

**Results:**

The study population had a mean gestational age of 39 weeks and 2 days (1.5 weeks) and birth weight 3528 g (668 g). There were 950 heart rate notations from each device, but heart rate was absent from the reference monitor in 14 of these data points, leaving 936 data pairs to compare. The mean (SD) difference when comparing NeoBeat to the reference monitor was -0.25 (9.91) beats per minute (bpm) (*p* = 0.44). There was a deviation of more than 10 bpm in 7.4% of the data pairs, which primarily (78%) was attributed to ECG signal disturbance, and secondly (22%) due to algorithm differences between the devices. Excluding these outliers, the correlation was equally consistent (r^2^ = 0.96) in the full range of heart rate captured measurements with a mean difference of − 0.16 (3.09) bpm. The mean difference was less than 1 bpm regardless of whether outliers were included or not.

**Supplementary Information:**

The online version contains supplementary material available at 10.1186/s13104-021-05576-x.

## Introduction

Approximately 5–6% of newborns require resuscitation at birth [1–3]. Heart rate is the most important clinical indicator used to assess the need for resuscitation and effectiveness of interventions [3].

**Fig. 1:**
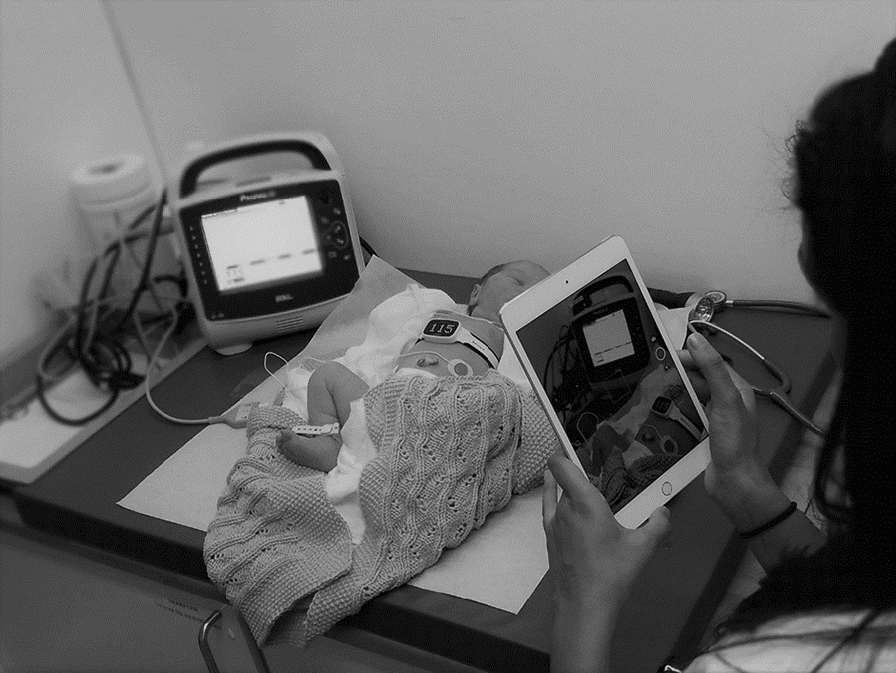
NeoBeat and reference monitor. Data collection of heart rate measurements by the dry-electrode NeoBeat and gel-electrode reference monitor

To obtain rapid and reliable heart rate monitoring during newborn resuscitation is challenging. Comparative studies show that electrocardiogram (ECG) provide feedback of heart rate earlier than pulse oximetry, and pulse oximetry can underestimate initial heart rate [4–9]. The International Liaison Committee on Resuscitation (ILCOR) suggests using ECG for accurate estimation of heart rate during newborn resuscitation, and emphasizes the importance of speed and reliablity [3, 10]. A novel, wireless, dry-electrode ECG device NeoBeat Newborn Heart Rate Meter (Laerdal Global Health, Stavanger, Norway) (Fig. 1) can be placed around the newborns wet torso or abdomen immediately after birth and display heart rate from 5 s after birth [11, 12]. Randomized controlled trials are ongoing to evaluate if immediate and continuous heart rate feedback during resuscitation of newborns by dry-electrode ECG technology improves compliance with guidelines and clinical outcomes. However, validation studies comparing heart rate measurements obtained by NeoBeat to conventional ECG from clinical settings are lacking. Does heart rate measurements obtained by the dry-electrode ECG device NeoBeat correlate with that of a conventional gel-electrode ECG monitor in healthy newborns?

## Objective

The aim of this study was to correlate the heart rate detection properties of a dry-electrode ECG device, NeoBeat (Laerdal Global Health), to that of a conventional 3-lead gel electrode ECG, PropaqM® (Zoll) in newborns.

## Main text

### Materials and methods

This single-centre observational study was conducted in the maternity unit at Stavanger University Hospital, Stavanger, Norway. Healthy newborns with birth weight ≥ 1500 g were included during their first day of life April 12th–May 26th 2019. Inclusion happened on random days when the investigators were on call and time permitted. Newborns in need of medical interventions at birth or admission in the Neonatal Intensive Care Unit were excluded. All parents of approached eligible newborns agreed to participate in the study.

The sample size was estimated using single population proportion formula with the assumption of proportion (p) for deviation score, p = 10%, with the standard deviation 95% level of certainty (2.5), and margin of error 0.1. This gave an estimate of 525 data points. An addition of 20% for missing data due to potential technical errors gave an estimate of 630 data points. We included a sample of 50 newborns in the analyses, sampled every 10 s for 3 min, resulting in a total of 950 data points from each device.

The wireless, dry-electrode ECG device NeoBeat (Laerdal Global Health, Stavanger, Norway) was developed for heart rate monitoring in newborns. The device can be rapidly applied to the newborns wet torso or abdomen after birth, and presents heart rate on a display on the sensor. For comparison, we used a reference monitor, a conventional 3-lead gel-electrode ECG (Propaq M, Zoll, Sydney, NSW, Australia) with BlueSensor ECG electrodes (Ambu, Ballerup, Denmark). In both devices, ECG biosensors and algorithms detect and display the heart rate based on QRS complex detection. An internal algorithm in each device designed by the manufacturer determines how QRS detection is transformed into heart rate displayed on the screen. Differences in this algorithm i.e. how many QRS complexes are used for averaging heart rate, will have an effect on the heart rate displayed.

During data collection, the newborns were placed in the supine position. The conventional 3-lead gel-electrode ECG was connected to the newborn’s torso as per the device’s instructions and the dry-electrode device attached across the torso. Electrodes of the two devices were located apart from each other (Fig. 1).

Heart rates displayed from the two devices were video-recorded for three minutes. Two researchers, HP and SR, reviewed the videos individually and registered heart rate from each device at 10-s intervals for 3 min, providing 19 notations per device per participant. The resulting 950 heart rate data points per device gave 950 data pairs to compare. To avoid human error in the notation process, discrepancies in the heart rate of more than five beats per minute (bpm) between the two researchers led to a re-examination of the video recordings and consensus on correct heart rate was reached. The notations from the two researchers were averaged for the subsequent analyses.

#### Statistical analysis

Data were analyzed with Matlab (The MathWorks, Natick, MA). Continuous variables were expressed as mean (standard deviation) unless otherwise stated. The devices were compared using a paired t-test. The device differences were illustrated using a correlation plot and a Bland–Altman plot. In analysis of the two devices, the conventional 3-lead gel-electrode ECG was the reference monitor for comparison. We defined outliers as ≥ 10 bpm rate difference between the devices and these were further analyzed to find the likely reason for the deviation, including re-examination of the video recordings.

### Results

The study population (n = 50) had a mean gestational age of 39 weeks and 2 days (1.5 weeks) and birthweight 3528 g (668 g). All data were checked with double entry and the inter-reviewer agreement correlated well (Pearsons’ r = 0.9993).

**Fig. 2:**
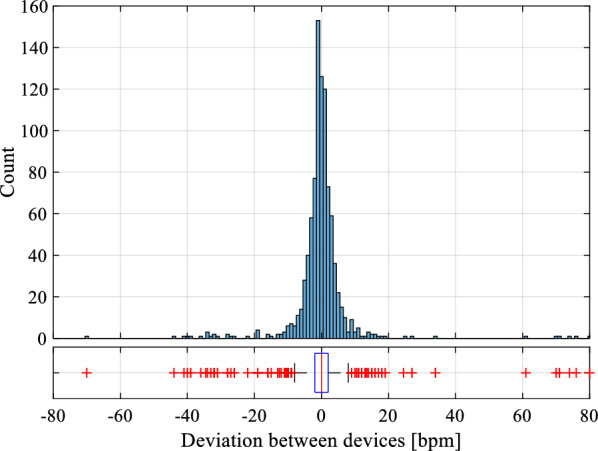
Deviation between the devices. Histogram and boxplot of deviation between devices. Y-axis indicate number of measurements and x-axis indicate the heart rate in beats per minute

There were 950 heart rate notations from each device, but heart rate was absent from the reference monitor in 14 of these data points, leaving 936 data pairs to compare. The mean (SD) difference when comparing NeoBeat to the reference monitor was − 0.25 (9.91) bpm (*p* = 0.44) (Fig. 2). Heart rate variability between the two devices over time in an individual newborn is presented in Additional file 1: Figure S1.

Assuming the heart rate of healthy newborns was stable over the three minutes of measurement, a linear fit of heart rate measurements was calculated for each measurement series. The mean square error between the data points and the linear fit was then calculated for each newborn for each device. The ensemble mean square error for NeoBeat and the reference monitor were 33.3 and 115.2 respectively. This shows that the reference monitor had more spurious measurements contributing to more deviation between the measurement devices.

#### Analysis of outliers

There were 69/936 data pairs (7.4%) with a deviation ≥ 10 bpm. Poor ECG signal in the reference monitor was noted as the reason in 54/69 (78%) data points. 30 of these 54 were because of ECG signal disturbance due to newborn movements (e.g. coughing, hiccupping, crying, spontaneous movements). The remaining data points were due to heart rate recovery thereof (i.e. when the monitor heart rate averaged back to “correct” heart rate).

In the reminding 15 of 69 data pairs (22%), newborn movements could not explain the deviations.

**Fig. 3:**
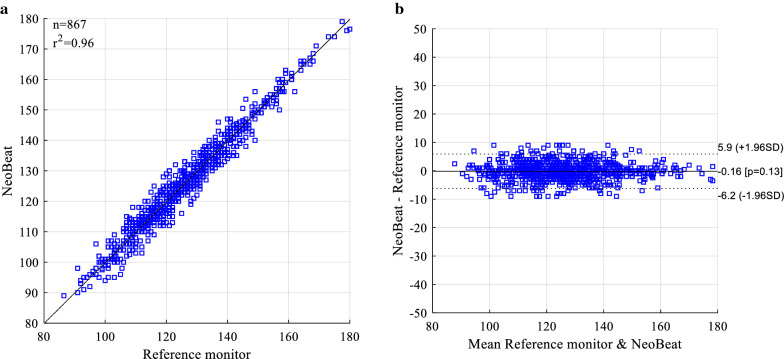
Correlation between devices. Correlation plot (**a**) and Bland–Altman plot (**b**) for the data set with the 69 outliers removed

When the 69 outliers were removed, the devices showed consistent correlation (r^2^ = 0.96) in the full range of captured heart rate measurements. The mean difference, when comparing the dry-electrode sensor to the reference monitor, excluding outliers was − 0.16 (3.09) bpm (Fig. 3).

### Discussion

In this study, we found no systematic errors or discrepancies between the two devices. The dry-electrode device showed a high degree of correlation with the reference ECG monitor, with less than 1 bpm difference. This finding was valid regardless of whether the outliers were included or not. The outliers observed were predominantly due to episodes of poor ECG signal in the reference monitor, mostly due to spontaneous movement of the newborns.

In 1.6% of the data points the discrepancies between the two devices were probably attributable to differences in the internal algorithm of the two devices. Heart rate frequency can be detected from ECG signal by different algorithms, based on QRS complex detection where heart rate is computed from the distance between the QRS complexes. We speculate that the reference monitor has a shorter averaging window for heart rate detection and presentation on the screen than NeoBeat. Although the systematic difference was minimal, heart rate from the 3-lead gel-electrode ECG monitor increased or decreased some seconds before heart rate from the dry-electrode device. This observed difference between the devices is clinically insignificant.

Our findings suggest that the reference monitor can be motion sensitive. This is important to consider in a resuscitation situation when interpreting the readings of any ECG device is crucial. A concern identified by ILCOR is initiated treatment based on a false positive low heart rate reading [10]. The dry-electrode device showed a lower sensitivity to motion and may be promising as a reliable device during resuscitation.

The findings of this study have important clinical implication demonstrating that heart rate measurements obtained by NeoBeat dry-electrode ECG correlate well with conventional gel-electrode ECG in healthy newborns. Correlation studies comparing accuracy of heart rate detection properties at low and high heart rates during newborn resuscitations are warranted.

### Conclusion

NeoBeat dry-electrode ECG was equally reliable and accurate over time as compared to a conventional 3-lead ECG device. The mean difference was less than 1 bpm regardless of whether outliers were included or not. Clinically insignificant, short-duration deviations were documented. These could be attributed to internal algorithm differences between the two devices.

## Limitations

A limitation of our study was that correlation analysis was performed in healthy newborns with heart rate measurements in the normal range 100–160 bpm, not during resuscitation when lower and higher heart rates would be expected (Additional file 2).

## Supplementary Information

**Additional file 1: Figure S1. **Heart rate variability between devices. **Figure S1.** Heart rate variability over time in an individual newborn.

**Additional file 2.** Heart rate data pairs from the two devices by the two observers.

## Data Availability

Data were stored and processed in a research database via an identification code. All data generated or analyzed during this study are included in this published article and its additional files.

## Abbreviations

bpm: Beats per minute
ECG: Electrocardiogram
ILCOR: The International Liaison Committee on Resuscitation

## References

[1] Bjorland PA, Oymar K, Ersdal HL, Rettedal SI (2019). Incidence of newborn resuscitative interventions at birth and short-term outcomes: a regional population-based study. BMJ Paediatr Open..

[2] Skare C, Kramer-Johansen J, Steen T, Odegaard S, Niles DE, Nakstad B (2015). Incidence of newborn stabilization and resuscitation measures and guideline compliance during the first minutes of life in Norway. Neonatology.

[3] Wyckoff MH, Wyllie J, Aziz K, de Almeida MF, Fabres J, Fawke J (2020). Neonatal life support: 2020 international consensus on cardiopulmonary resuscitation and emergency cardiovascular care science with treatment recommendations. Circulation.

[4] van Vonderen JJ, Hooper SB, Kroese JK, Roest AA, Narayen IC, van Zwet EW (2015). Pulse oximetry measures a lower heart rate at birth compared with electrocardiography. J Pediatr..

[5] Mizumoto H, Tomotaki S, Shibata H, Ueda K, Akashi R, Uchio H (2012). Electrocardiogram shows reliable heart rates much earlier than pulse oximetry during neonatal resuscitation. Pediatr Int..

[6] Bjorland PA, Ersdal HL, Oymar K, Rettedal SI (2020). Compliance with guidelines and efficacy of heart rate monitoring during newborn resuscitation: a prospective video study. Neonatology.

[7] Murphy MC, De Angelis L, McCarthy LK, O'Donnell CPF (2019). Randomised study comparing heart rate measurement in newly born infants using a monitor incorporating electrocardiogram and pulse oximeter versus pulse oximeter alone. Arch Dis Child Fetal Neonatal Ed..

[8] Iglesias B, Rodri Guez MAJ, Aleo E, Criado E, Marti Nez-Orgado J, Arruza L (2018). 3-lead electrocardiogram is more reliable than pulse oximetry to detect bradycardia during stabilisation at birth of very preterm infants. Arch Dis Child Fetal Neonatal Ed..

[9] Gulati R, Zayek M, Eyal F (2018). Presetting ECG electrodes for earlier heart rate detection in the delivery room. Resuscitation.

[10] Perlman JM, Wyllie J, Kattwinkel J, Wyckoff MH, Aziz K, Guinsburg R (2015). Part 7: neonatal resuscitation: 2015 international consensus on cardiopulmonary resuscitation and emergency cardiovascular care science with treatment recommendations. Circulation.

[11] Linde JE, Schulz J, Perlman JM, Oymar K, Francis F, Eilevstjonn J (2016). Normal newborn heart rate in the first five minutes of life assessed by dry-electrode electrocardiography. Neonatology.

[12] Bjorland PA, Ersdal HL, Eilevstjonn J, Oymar K, Davis PG, Rettedal SI (2020). Changes in heart rate from 5 s to 5 min after birth in vaginally delivered term newborns with delayed cord clamping. Arch Dis Child Fetal Neonatal Ed..

