# Supplementary figures and images for: Heart rate detection properties of dry-electrode ECG compared to conventional 3-lead gel-electrode ECG in newborns
Source: BMC Res Notes. 2021 May 1;14:166. doi: 10.1186/s13104-021-05576-x (PMC8088562; doi:10.1186/s13104-021-05576-x)

**11. MSE NeoBeat = 17.28. MSE Reference monitor = 126.31**

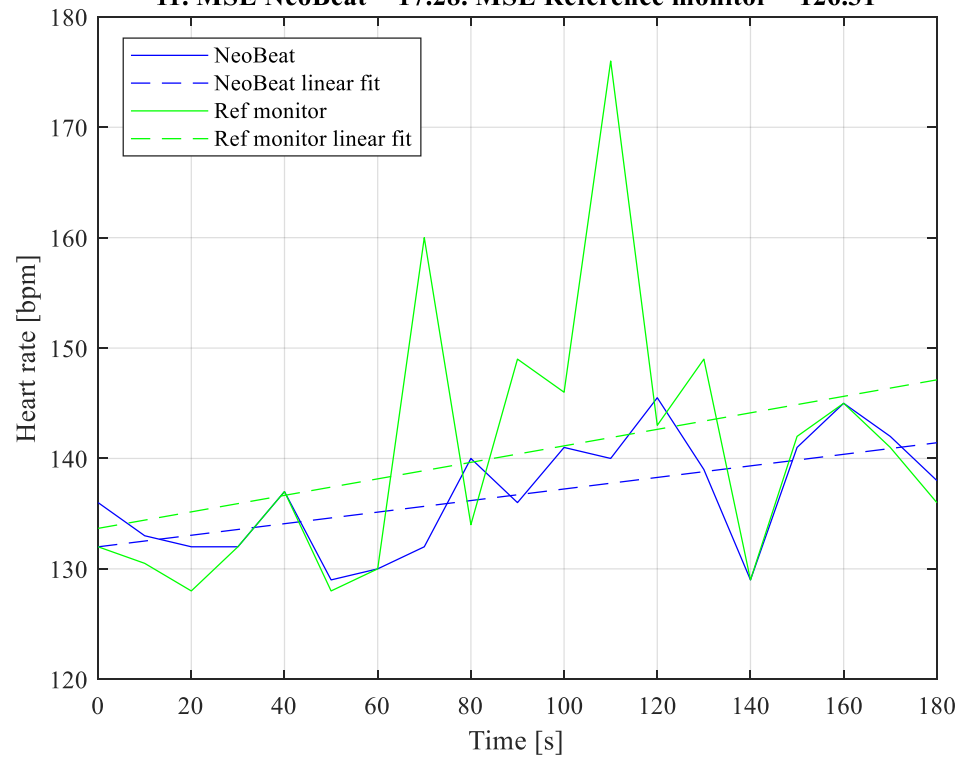

Supplement: Supplementary file 1 — Additional file 1: Figure S1. Heart rate variability between devices. Figure S1. Heart rate variability over time in an individual newborn. [file 13104_2021_5576_MOESM1_ESM.pdf]
